# Supplementary material for: Probing the limits of effective temperature consistency in actively driven systems
Source: Soft Matter. 2025 Dec 15;22(2):297–305. doi: 10.1039/d5sm00840a (PMC12771276; doi:10.1039/d5sm00840a)
Supplement: SM-022-D5SM00840A-s001 [file SM-022-D5SM00840A-s001.pdf]

Supplementary Information (SI) for Soft Matter.
This journal is © The Royal Society of Chemistry 2025

Probing the limits of effective temperature consistency in actively driven systems

Dima Boriskovsky,[1] Rémi Goerlich,[2] Benjamin Lindner,[3,4] and Yael Roichman[1,2,*]

[1] *Raymond & Beverly Sackler School of Physics and Astronomy, Tel Aviv University, Tel Aviv 6997801, Israel*
[2] *Raymond & Beverly Sackler School of Chemistry, Tel Aviv University, Tel Aviv 6997801, Israel*
[3] *Bernstein Center for Computational Neuroscience Berlin, Philippstr. 13, Haus 2, 10115 Berlin, Germany*
[4] *Physics Department of Humboldt University Berlin, Newtonstr. 15, 12489 Berlin, Germany*

SUPPLEMENTARY INFORMATION

Further results and measurements

We provide the following supplemental material: The average force exerted by the fan on the tracer was measured at different distances under a constant air stream (without bbots). The variance and skewness of the tracer's stationary distributions (with $N_b = 10$) are presented for different air stream amplitudes, corresponding to varying fan voltages (Fig. 1); FDR tests under constant N_b and increasing perturbation amplitudes (Fig. 2); Individual FDR results for position and velocity tracer observables (Figs. 3,4) and work FR results (Fig. 5), with different N_b. Particularly, these results were obtained with the *standard* experimental setup, as detailed in the main paper.

Fig. 6 and 7 present FDR and FR tests with alternative system configurations. Namely, we employ faster bbots and a smaller styrofoam ball as a passive tracer, respectively. The position autocorrelation functions are approximately proportional to the responses with $T_{\text{eff}} = T_{\text{pot}}$, yet the FDR in the velocity observable show violations for short times. The work FR is valid for sufficiently small values, from which a consistent effective temperature can be extracted with $T_{\text{FR}} \approx T_{\text{eff}}$. These measurements fail with a heavy tracer setup (Fig. 8), as discussed in the main text.

We note that additional reliable T_{eff} measurements across different N_b would require additional tuning of the perturbation amplitude and the potential stiffness, to ensure stable dynamics in the system.

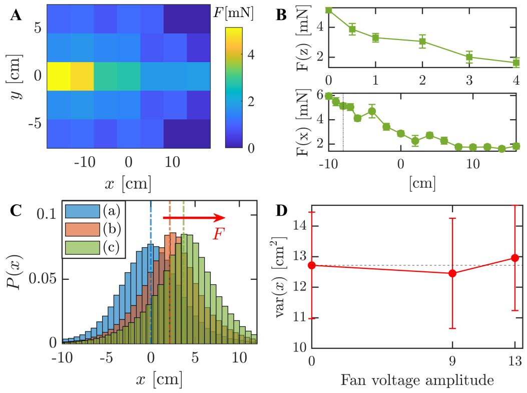

FIG. 1. **Forces and stationary statistics under external air stream. A, B.** Average forces acting on the tracer in the absence of bbots, on a flat surface. Measurements were obtained using a force sensor (Mark-10 Force Gauge) with 10V operating fan voltage: (A) as a function of $x-y$ position; (B) along the main air stream axis x (lower panel) - and as a function of height z (upper panel), at a distance $x = 8$cm marked by the vertical dashed line. Error bars are standard deviations of 50 measurements. **C.** Stationary probability distributions of the tracer's position under different air stream intensities in the harmonic trap (unperturbed, 10V, and 13.5V fan operating voltage), with $N_b = 10$. **D.** Corresponding position variances. Results were obtained from time and ensemble averages over $M = 375$ realizations of one-minute tracer trajectories. Error bars indicate standard deviations.

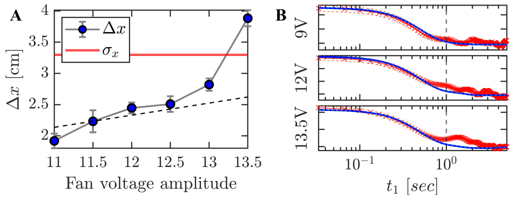

FIG. 2. **The FDR test under different perturbation amplitudes. A.** The mean displacement Δx under a perturbation of a tracer in a $N_b = 10$ bbot bath, as a function of fan operating voltage V (error bars are standard errors). The applied force $F_0 = k\Delta x$ increases above $V = 11$V. The tracer's position standard deviation, σ_x, is used to define the range of small perturbations (solid line). The dashed line is a linear fit of the first 4 data points, with $\Delta x = 0.19 \cdot V$, whereas $V = 13$V and 13.5V are out of the linear regime. **B.** The generalized FDRs with $T_{\text{eff}} \sim \langle \Delta x^2 \rangle_0$ are plotted for $V = 9,\ 12,\ 13.5$ V, with $N_b = 10$. The results present an average over $M = 375$ perturbation sequences.

* roichman@tauex.tau.ac.il

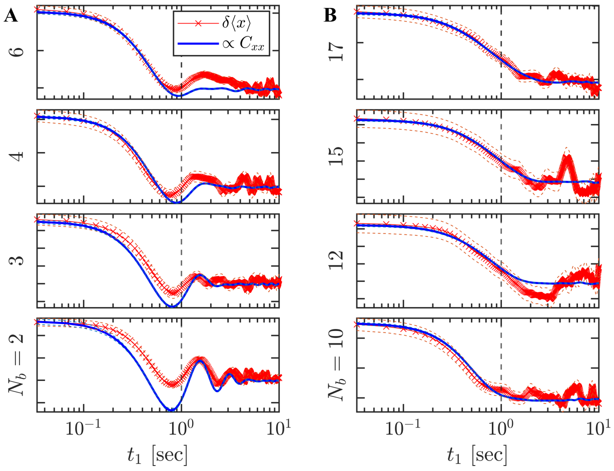

FIG. 3. **Position (x) FDR results with different numbers of bbots. A,B.** FDRs with $T_{\text{eff}} \sim \langle \Delta x^2 \rangle_0$ for $N_b = \{2, 3, 4, 6, 10, 12, 15, 17\}$ bbot baths. The results were obtained for an ensemble average of $M = 375$ perturbation sequences, with the same tracer $m \approx 1$ g, gravitational stiffness $k \approx 28.2$ g/s^{-2}, and fan operating voltage (9 V) with $\Delta x_\epsilon < \sigma_x$. The vertical dashed lines are $T_c = 1$ s.

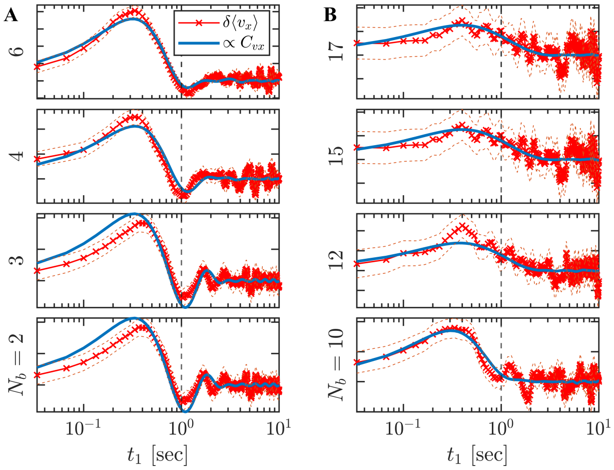

FIG. 4. **Velocity (v_x) FDR results with different numbers of bbots. A,B.** FDRs with $T_{\text{eff}} \sim \langle \Delta x^2 \rangle_0$ for $N_b = \{2, 3, 4, 6, 10, 12, 15, 17\}$ bbot baths. Same details as in Fig. 3.

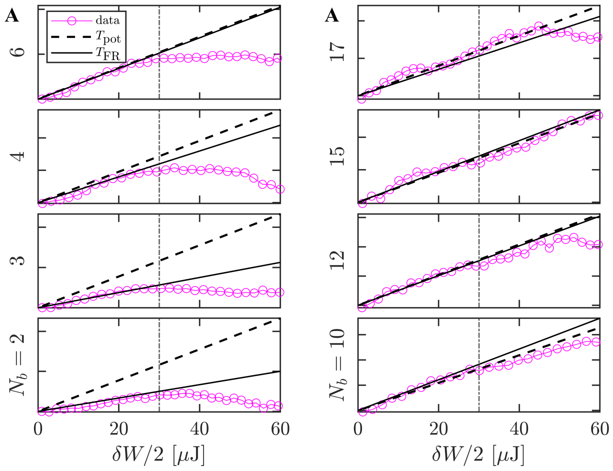

FIG. 5. **Work FR results in (strongly) perturbed steady-states with different numbers of bbots. A,B.** Work FRs with $N_b = \{2, 3, 4, 6, 10, 12, 15, 17\}$. The solid line represent a linear fit with a slope of $\sim 1/T_{\text{FR}}$. The dashed line has a slope of $\sim 1/T_{\text{pot}}$. The results were obtained for an ensemble average of $M = 375$ perturbation sequences, with the same tracer $m \approx 1$ g, gravitational stiffness $k \approx 28.2$ g/s^{-2}, and fan operating voltage of 13.5 V.

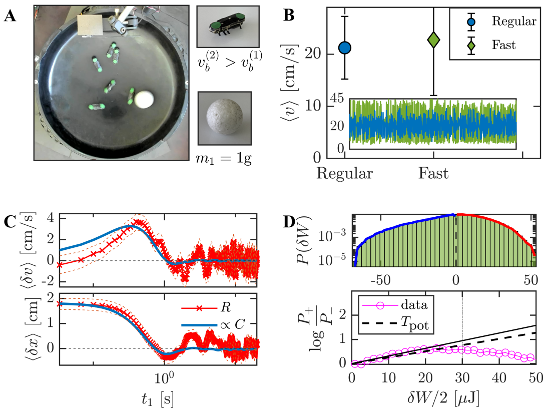

FIG. 6. **Fast bbot setup.** **A.** The active bath contains $N_b = 6$ *fast* bbots, the experimental setup is otherwise the same as in Figs. 2-7. **B.** Average individual speed of a single bbot walking in a parabolic arena. Standard bbots (circle) and fast bbots (diamond), as referred to in the main paper. **C.** FDR test for the tracer's position and velocity observables, under the same perturbation setup as detailed in Figs. 3,4. The effective temperature is $T_{\rm eff} = \kappa \langle x^2 \rangle_0$. **D.** Work FR test as detailed in Fig. 5.

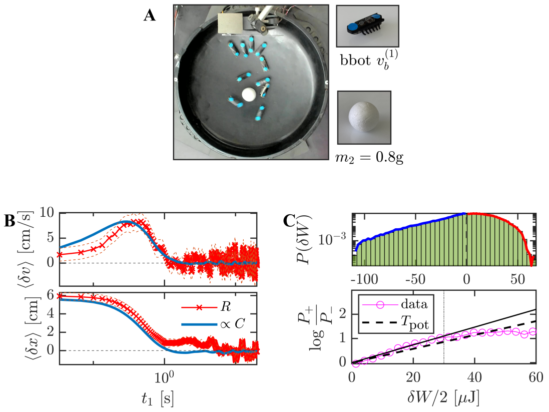

FIG. 7. **Small tracer setup.** **A.** A small styrofoam ball is used as a passive tracer (3 cm diameter, 0.8 g). The active bath contains $N_b = 10$ standard bbots. **B.** FDR test for the tracer's position and velocity observables, under the same perturbation setup as detailed in Figs. 3,4. The effective temperature is $T_{\rm eff} = \kappa \langle x^2 \rangle_0$. **C.** Work FR test as detailed in Fig. 5.

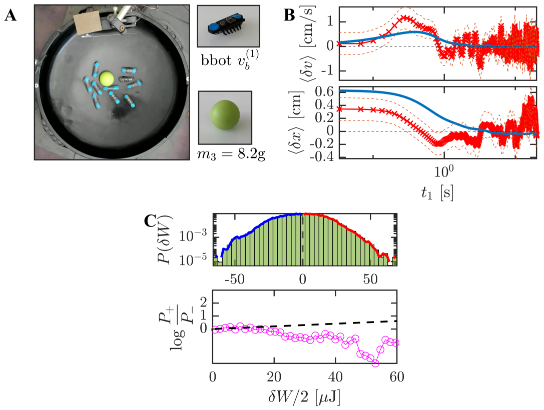

FIG. 8. **Heavy tracer setup.** **A.** A heavy plastic ball is used as a passive tracer (3 cm diameter, 8.2 g). The active bath contains $N_b = 10$ standard bbots. **B.** FDR test for the tracer's position and velocity observables, under the same perturbation setup as detailed in Figs. 3,4. A strong airflow (13.5 V fan operating voltage) was used as a step-perturbation. The effective temperature is $T_{\rm eff} = \kappa \langle x^2 \rangle_0$. The correlation functions fail to capture the response to the step-perturbation. **C.** Work FR test as detailed in Fig. 5, does not yield an effective temperature.

System technicalities

In our experimental setup, the number of bbots (N_b) is limited due to their tendency to occasionally tumble upon collisions with other bbots within the potential well. This tumbling effect becomes increasingly pronounced at high densities (and high propulsion speeds), leading to the formation of long-lived static clusters at the trap center. Such clustering can prevent the tracer from accessing certain regions in the trap bulk, thus hindering reliable measurements of the its fluctuations and responses. This effect can be partly observed in the stationary (tracer) probability distribution with $N_b = 17$, as shown in Fig. 9.

In Fig. 10 we present measurements that quantify the chirality of a single bbot in an assembly composed of N_b bbots (in the potential well). We evaluate the net chirality of a system by counting the clockwise (+1) and anticlockwise (−1) crossings (across a central line at $x = 0$). The results were obtained by tracking 200 bbot trajectories ($t \in [0, 5]$ s), and present a sum of $\pm n$ rotations per frame, and a probability distribution $P(n)$. We find that the systems attain a net clockwise chirality that is reduced by increasing N_b, and therefore the collision frequency. In comparison, the chirality of the passive tracer particle results in small values and vanish for sufficiently high N_b.

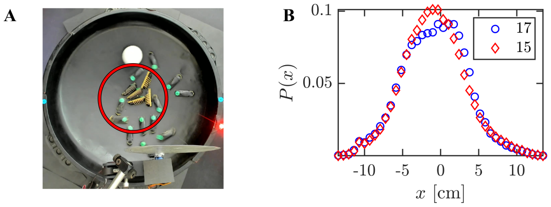

FIG. 9. **A.** Snapshot of an experiment with $N_b = 17$, showing a temporal static cluster of tumbled bbots in the center trap region. **B.** The tracer's position probability distribution for $N_b = 17$, showing a flat region in the center, reflecting the physical exclusion by static bbot clusters.

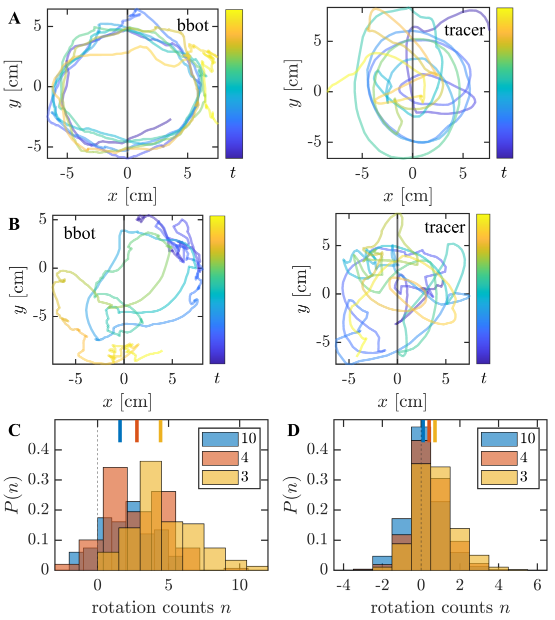

FIG. 10. **The system's chirality.** Typical single bbot and tracer trajectories, in a system with $N_b = 3$ (**A**) and $N_b = 10$ (**B**). **C.** The distribution of bbot instantaneous rotations $\pm n$ across a central line at $x = 0$, where $n > 0$ indicates favored clockwise direction. Obtained by tracking 200 bbots trajectories ($t \in [0, 5]s$). The upper lines are the means of $P(n)$, showing that net chirality is reduced by increasing N_b. **D.** The same chirality analysis for the passive tracer.
