# Supplementary material for: Probing the limits of effective temperature consistency in actively driven systems
Source: Soft Matter. 2025 Dec 15;22(2):297–305. doi: 10.1039/d5sm00840a (PMC12771276; doi:10.1039/d5sm00840a)
Supplement: SM-022-D5SM00840A-s004 [file SM-022-D5SM00840A-s004.pdf]

Supplementary Information (SI) for Soft Matter.
This journal is © The Royal Society of Chemistry 2025

Supplemental Movie 1 Description:

Experimental recordings that display the motion of the self-propelled bbots and the driven tracer particle within the gravitational harmonic arena. For sufficiently large number of bbots N_b, the active medium behaves like an active gas-like state, frequently and randomly colliding with the passive tracer, thereby driving a Brownian-like motion in the potential well. The video shows the tracer motion under different N_b configurations.

Supplemental Movie 2 Description:

The video was recorded using an ultra-high-speed camera operating at 6400fps, capturing a typical experimental time interval of $\Delta t \approx 0.03$s used to track tracer trajectories. The footage compares the motion of a light versus a heavy tracer within an active system of $N_b = 6$ self-propelled bbots.

The heavy tracer can undergo a sequence of persistent, directed collisions with the bbots, which are coarse-grained at the experimental resolution of Δt. During strong impacts, particularly at higher relative velocities, the heavy tracer is capable of altering the bbots' dynamics, noticeably affecting their post-collision velocities. Over longer timescales, the video shows that the bbots can transiently "cage" the heavy tracer, causing it to remain confined within a cluster in the trap.

In contrast, the light tracer exhibits ballistic motion between discrete collision events, each acting as a distinct "kick". Unlike the heavy tracer, the light tracer does not typically become caged and remains free from clustering effects throughout its motion in the system.
